# Fructose-1,6-bisphosphatase loss modulates STAT3-dependent expression of PD-L1 and cancer immunity

**DOI:** 10.7150/thno.38137

**Published:** 2020-01-01

**Authors:** Bo Wang, Yingke Zhou, Jun Zhang, Xin Jin, Heshui Wu, Haojie Huang

**Affiliations:** 1 Department of Pancreatic Surgery, Union Hospital, Tongji Medical College, Huazhong University of Science and Technology, Wuhan, 430022, China;; 2 Department of Biochemistry and Molecular Biology, Mayo Clinic College of Medicine and Science, Rochester, MN 55905, USA.; 3 Department of Digestive Surgical Oncology, Union Hospital, Tongji Medical College, Huazhong University of Science and Technology, Wuhan, 430022, China.; 4 Department of Laboratory Medicine and Pathology, Mayo Clinic College of Medicine and Science, Rochester, MN 55905, USA;; 5 Department of Urology, Mayo Clinic College of Medicine and Science, Rochester, MN 55905, USA;; 6 Mayo Clinic Cancer Center, Mayo Clinic College of Medicine and Science, Rochester, MN 55905, USA.

**Keywords:** PD-L1, FBP1, STAT3, cancer immunity, checkpoint blockade therapy

## Abstract

**Rationale**: Abnormal expression of programmed death-1 (PD-1) ligand-1(PD-L1) in cancer cells plays a crucial role in cancer immune evasion and progression. The immune checkpoint molecules PD-1 and PD-L1 have been targeted for cancer treatment with significant benefits for cancer patients. However, the response rate is relatively low in certain types of cancer and the underlying mechanism remains poorly understood. Better understanding of the molecular mechanism of PD-L1 expression regulation in cancer cells is urgently needed to improve the treatment response rate and overall survival of patients. Fructose-1, 6-biphosphatase (FBP1) is a key enzyme in gluconeogenesis and is implicated in human cancer due to its frequent loss in various cancer types.

**Methods**: Expression of FBP1 and PD-L1 was analyzed in various cancer cell lines. Western blot and RT-qPCR were performed to determine whether FBP1 regulates PD-L1 expression. Co-immunoprecipitation and glutathione S-transferase (GST) pulldown assay were employed to define the underlying regulatory mechanisms. Immunohistochemistry was conducted to determine the correlation between FBP1 and PD-L1 expression in a cohort of patients. A cancer syngeneic mouse model was utilized to examine how FBP1 affects tumor immunity.

**Results**: We demonstrated that in a manner independent of its enzymatic activity FBP1 downregulates the expression of PD-L1 in various cell lines of different cancer types including pancreatic and prostate cancer. We further showed that this regulation occurs at the transcriptional level and is mediated by FBP1 inhibition of signal transducer and activator of transcription-3 (STAT3)-dependent PD-L1 transcription. Moreover, FBP1 and PD-L1 protein expression were negatively correlated in pancreatic ductal adenocarcinoma (PDAC) specimens from a cohort of patients. Most importantly, we demonstrated that decreased FBP1 expression promotes tumor growth and resistance to immune checkpoint blockade therapy in mice.

**Conclusions:** Our findings reveal a new tumor suppressor function of FBP1 in inhibiting PD-L1 expression and enhancing cancer immunity. They also suggest that FBP1-deficient human cancers could be therapeutically targeted by PD-1/PD-L1-based immune checkpoint blockade therapy.

## Introduction

Immune evasion is one of the key oncogenesis hallmarks of human cancers 1. While the neonatal antigens derived from solid tumor remain capable of triggering cancer immunity, tumors often escape from immune attack. Recent studies have shown that immune evasion of cancers can be achieved by many mechanisms, including the abnormal upregulation of immune checkpoint molecules 2.

Programmed death-1 (PD-1) and its ligand PD-L1 are two well-studied checkpoint molecules 3-5. PD-L1 expressed in cancer cells binds to PD-1 in T cells and induces inactivation and/or apoptosis of T cells, resulting in the immune escape of cancer 3-6. Existent studies have shown that PD-L1expression in cancer cells is regulated by various mechanisms. Up-regulation of PD-L1 in cancer cells heavily depends on the activation of TLR- or IFN-γ-mediated signaling pathways, such as NF-κB, MAPK, PI3K, mTOR and JAK. Activation of these signaling cascades results in nuclear translocation of numerous transcription factors that transactivate expression of *PD-L1* gene 7-11. These transduction pathways can be activated by pro-inflammatory cytokines such as IFN-γ, TNF-α, IL-1β or due to loss or inactivation of tumor suppressor genes such as *PTEN* and *RB* 12, 13. Additionally, PD-L1 levels can also be upregulated due to deregulated proteolytic degradation of PD-L1 protein 14, 15.

Anti-PD1 and anti-PD-L1 antibodies are utilized for cancer treatment in patients. FDA has approved anti-PD therapy for at least 11 cancers including metastatic melanoma, lung cancer, renal cell carcinoma urothelial carcinoma, gastric cancer, liver cancer, Hodgkin's lymphoma and so on 16. Existent data have shown that PD-1/PD-L1 immune checkpoint-based therapy has significantly improved progress-free survival in a subset of patients, especially in carcinogen-induced cancers or cancers caused by viral infections. Hodgkin's lymphoma 17, desmoplastic melanoma 18 and virials induced Merkel cell carcinoma are cancer indications with highest response rate (50-90%) 19. However, the response rate is relatively low in certain cancer types 20-23. The most common reasons of the low response rate are rare T cell infiltration in tumor or impaired antitumor T cells by other checkpoints, such as CTLA-4 or immune suppressive cells in tumor microenvironment or cancer cells insensitive to IFN-γ leading to no or low reactively expressed PD-L1 24-27. Thus, better understanding of the molecular mechanism that regulates PD-L1 expression in cancer cells is of significant importance to improve patient response to anti-PD-1/PD-L1 therapy in clinic.

Fructose-1, 6-biphosphatase (FBP1) primarily functions as a negative regulator of glycolysis. Increasing evidence suggests that it possesses a tumor suppressor function and it is often downregulated in many types of human cancer 28. Loss of FBP1 is associated with tumor progression and patient prognosis in various cancer types examined 28-30. Previous studies have showed that FBP1 suppresses the aggressive features of hepatocellular carcinoma cells through regulating the Warburg effect or modulating the metabolism of breast cancer cells via inhibition of expression of hypoxia inducible factor 1α (HIF-1α) 30, 31. In the present study, we identified a role of FBP1 in suppressing the expression of PD-L1 and this effect is mediated by its interaction with STAT3. Our findings also suggest that FBP1 loss may be a pervasive mechanism that contributes to the deregulation of PD-L1 in various human cancers.

## Materials and Methods

### Cell lines and cell culture

Prostate and breast cancer cell lines VCaP, PC-3, MCF-7, T47D and MDA-MB-231 were purchased from ATCC. PTEN-CaP8 murine Pten-deficient CRPC cell line was kindly provided by Dr. Hong Wu at UCLA 32. The pancreatic cancer cell lines PANC-1 and MIA PaCa-2 were obtained from Dr D.D. Billadeau at Mayo Clinic (Rochester, MN). Cell lines were cultured in DMEM or RPMI supplied with 10% fetal bovine serum (FBS) and routinely maintained at 37 ℃, 5% CO2 incubator.

### Plasmids and antibodies

Flag-FBP1, HA-FBP1 and Flag-STAT3 expression vectors were generated using the pCMV backbone plasmid. The FBP1 mutant G260R and STAT3 mutant Y705D were generated using KOD-Plus Mutagenesis Kit (Toyobo). Antibodies used in this study were purchased from the following companies: FBP1 (Abcam); PD-L1, STAT3 and p-STAT3 (Y705) (Cell Signaling Technology); ERK2 (Santa Cruz Biotechnology); HA tag (Covance), and Flag tag (Sigma).

### Western blot

Cells were harvested and lysed in cell lysis buffer on ice for more than 15 minutes and the lysate was centrifuged at 13,000 rpm at 4 ℃ for 10 minutes. The supernatant was transferred into a new tube for BCA protein quantification assay (Thermo Fisher Scientific). 4x loading buffer (Thermo Fisher Scientific) was added into equal amount of protein sample and boiled for 5 minutes. The sample was used for SDS-PAGE analysis and transferred to nitrocellulose membrane. The membrane was blocked with 5% non-fat milk in room temperature for 1 hour and incubated with primary antibody at 4 ℃ overnight. The membrane was washed with 1x TBST 5 minutes for 3 times, and incubated with horseradish peroxidase-conjugated secondary antibody for 1 hour in room temperature. The protein was visualized by SuperSignal West Pico Stable Peroxide Solution (Thermo Fisher Scientific).

### Real-time reverse transcription-polymerase chain reaction (RT-PCR)

Total RNA was extracted from cells using Trizol reagent (Thermo Fisher Scientific). First strand cDNA was synthesized from 1 µg of total RNA using the cDNA Reverse Transcription Kit. Real-time PCR analysis was performed according to the guidance of manufacturer's protocol. Sequences of primers for RT-qPCR are provided in Supplementary Table 1.

### Co-immunoprecipitation (co-IP)

Cells were harvested and lysed with IP buffer on ice for more than 15 minutes and the lysate was centrifuged at 13,000 rpm at 4 ℃ for 10 minutes. The supernatant was transferred into a new tube. The supernatant was incubated overnight with protein A/G agarose beads (Thermo Fisher Scientific) and primary antibodies under 4 ℃ with mild rocking. After incubation, the beads were washed 6 times with IP buffer on ice and then subjected into western blotting analysis.

### Tissue microarray (TMA) and immunohistochemistry (IHC)

TMA slides of pancreatic ductal adenocarcinomas (PDACs) were purchased from Biomax US (Catalog no. PA1001a). The specimens were immunostained with FBP1, PD-L1 and p-STAT (Y705) antibodies. Staining intensity was graded in a blinded fashion: 1 = weak staining at 100x magnification but little or no staining at 40x magnification; 2= medium staining at 40x magnification; 3= strong staining at 40x magnification. The final staining index was calculated by the formula: staining intensity x staining percentage.

### Glutathione S-transferase (GST) pulldown assay

Cells were lysed with cell lysis buffer (20 mM Tris-HCl (pH=7.5), 150 mM NaCl, 0.1% Nonidet P40, 1 mM dithiothreitol, 10% glycerol, 1 mM EDTA, 2.5 mM MgCl2 and 1 µg/ml leupeptin) for 30 minutes at 4 ℃. The GST fusion proteins were immobilized on glutathione-Sepharose beads (GE Healthcare Lifesciences), and the beads were washed with lysis buffer. The beads were incubated with cell lysate for 4 hours, and were washed four times with binding buffer and re-suspended in sample buffer. The bound proteins were analyzed by SDS/PAGE and western blot.

### RNA interference

Lentivirus-based control and gene-specific small hairpin RNAs (shRNAs) were purchased from Sigma-Aldrich. Lipofectamine 2000 was used to transfect 293T cells with shRNA plasmids and viral packaging plasmids (pVSV-G and pEXQV). At 24 hours after transfection, the medium was replaced with fresh DMEM, containing 10% FBS and 1 mM of sodium pyruvate. 48 hours after transfection, the virus culture medium was collected, filtered and added to cancer cells supplemented with 12 µg/mL of polybrene. At 24 hours after infection, the infected cells were selected with 10 µg /mL of puromycin. The shRNA and sgRNA sequence is provided in the Supplementary Table 2.

### Glucose consumption measurement assay

Cells were plated in 6-well plates and cultured in DMEM medium without phenol red (Thermo Fisher Scientific). 24 hours after plasmid transfection or 48 hours after lentivirus infection, the spent medium was collected. Glucose concentration of the spent medium was measured using a Glucose (GO) Assay Kit according to the manufacturer's instructions (Sigma-Aldrich). Glucose consumption was calculated by the difference of glucose concentration between the spent medium and unused medium.

### Generation of a prostate cancer syngeneic mouse model for anti-PD-L1 antibody treatment

C57BL/6 male mice at 6-week old were purchased from Jackson Laboratory. The animal study was approved by the Mayo Clinic IACUC. All mice were housed in standard conditions with 12-hour light/dark cycle and access to food and water ad libitum*.* PTEN-CaP8 murine prostate cancer cells (5×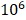 ) infected with lentivirus expressing control or Fbp1-specific shRNAs was injected subcutaneously into the right flank of mice. The volume of allografts was measured every other day until the tumor volume reached 300 mm^3^ and calculated by the formula (L × W2 × 0.5). At the end of measurement, mice were euthanized and tumors were isolated and weighed.

### Flow cytometry analysis

PANC-1 and MIA PaCa-2 cells infected with shRNA were harvested and washed with 1× PBS. Cells were fixed with 4% paraformaldehyde for 15 minutes. Cells were incubated with ice-cold 100% methanol for 30 minutes on ice followed by wash with 1× PBS. Cells were washed with 1× PBS one more time and incubated with antibody or isotype IgG for 1 hour at room temperature. Cells were incubated with secondary antibody conjugated with Alexa Fluor (Thermo Fisher Scientific) for 1 hour at room temperature followed by wash with 1× PBS. After washed three times with 1× PBS, cells were resuspended with 1× PBS and analyzed using flow cytometer.

For the preparation of flow cytometry analysis of mouse tissue samples, tumors were cut into small pieces and digested with 2 mg/ mL collagenase (Sigma Aldrich) in DMEM for 1 hour at 37 ℃. Cells were filtered through 70 µm nylon strainer and resuspended in red blood cell lysis buffer (Biolegend) for 3 minutes at room temperature. Cells were suspended in 1× PBS with 2% BSA and co-stained with antibodies. After incubated with antibody for 30 minutes, cells were washed with 1× PBS and analyzed with flow cytometer.

### Statistical analysis

Statistical analysis were carried out by one-sided or two-sided paired Student's t-test for single comparison and one-way ANOVA with a post-hoc test for multiple comparisons, and *P* values < 0.05 was considered statistically significant. All the values are expressed as the means ± SD.

## Results

### FBP1 negatively regulates PD-L1 expression in multiple cell lines of different cancer types

**Figure 1:**
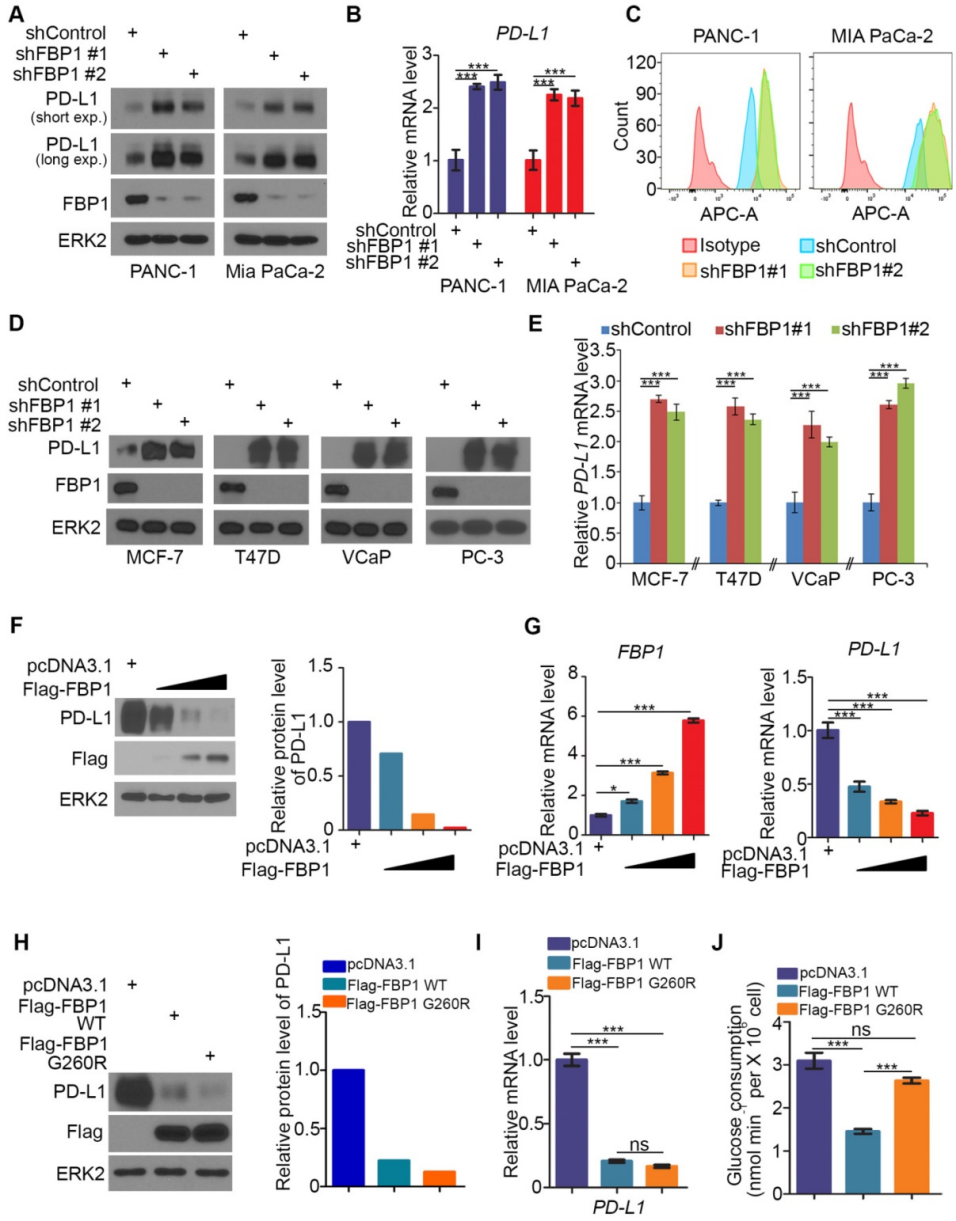
** FBP1 negatively regulates PD-L1 expression in multiple cancer cell lines.** (A-C) PANC-1 and MIA PaCa-2 cells were infected with lentivirus expressing control shRNA or FBP1-specific shRNA. After 48 hours of puromycin selection, cells were harvested for western blot (A), quantitative RT-PCR (B) and FACS (C). All data shown are mean values ± SD (n=3), *** *P* < 0.001. (D, E) MCF-7, T47D, VCaP and PC-3 cells were harvested for western blot and quantitative RT-PCR at 48 hours after infected with lentivirus expressing control shRNA or FBP1-specific shRNA. All data shown are mean values ± SD (n=3), *** *P* < 0.001. (F, G) PANC-1 cells were infected with control or FBP1 expressing plasmid. 24 hours later cells were harvested for western bolt (F) and quantitative RT-PCR (G). All data shown are mean values ± SD (n=3), * *P* < 0.05, *** *P* < 0.001. (H-J) PANC-1 cells were infected with indicated plasmids. After 24 hours cells were harvested for western blot (H) and quantitative RT-PCR (I). Spent medium was collected for measurement of glucose consumption (J). All data are shown as mean values ± SD (n=3). ns, not significant, *** *P* < 0.001.

It has been shown previously that FBP1 is frequently lost in many types of human cancers including renal carcinoma, basal-like breast cancer, hepatocellular carcinoma and pancreatic cancer and that loss of FBP1 promotes cancer progression, metabolic reprogramming and drug resistance 28, 31, 33, 34. Given that PD-L1 is a key immune checkpoint molecule and it is often deregulated in human cancers 3-5, 15, 35, we sought to determine whether FBP1 expression influence cancer immunity by regulating PD-L1 expression in cancer cells. To this end, we knocked down endogenous FBP1 using two independent shRNAs in PANC-1 and MIA PaCa-2 pancreatic cancer cell lines. FBP1 knockdown (KD) invariably increased expression of PD-L1 at both protein and mRNA levels as demonstrated by western blot and quantitative RT-PCR (Figures 1A and 1B). These results are consistent with increased expression of PD-L1 on the surface of FBP1 KD cells as demonstrated by FACS (Figure 1C). Similar results were observed in breast cancer cell lines MCF-7 and T47D and prostate cancer cell lines VCaP and PC-3 (Figures 1D and 1E). Accordingly, overexpression of FBP1 decreased PD-L1 expression at both protein and mRNA level in a dose dependent manner (Figures 1F and 1G). These data indicate that FBP1 has an inhibitory effect on the expression of PD-L1 in multiple cancer cell lines.

FBP1 is the key enzyme that inhibits glycolysis. To determine whether enzyme activity is required for FBP1 to inhibit the expression of PD-L1, a catalytically inactivated mutant of FBP1 (G260R) was constructed as described 36, 37. FBP1-WT and FBP1-G260R plasmids were transfected into PANC-1 cells. We demonstrated that ectopic expression of FBP1-WT and FBP1-G260R resulted in a similar inhibitory effect on the expression of PD-L1 at both protein and mRNA level (Figures 1H and 1I). As expected, ectopic expression of FBP1-WT, but not FBP1-G260R, inhibited the glucose consumption in these cells (Figure 1J). Our data indicates that FBP1 inhibits the expression of PD-L1 in a manner independent of its enzyme activity.

### FBP1 and PD-L1 expression are negatively correlated in PDAC patient specimens

**Figure 2:**
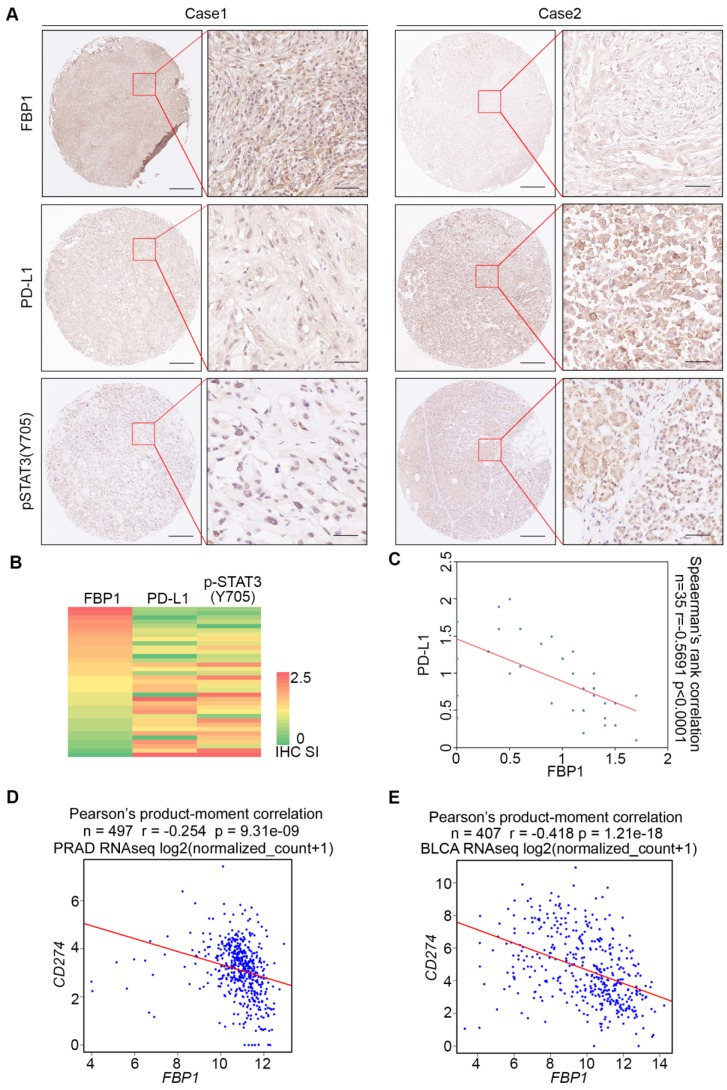
** FBP1 and PD-L1 expression are negatively correlated in PDAC patient specimens.** (A) Representative images of IHC of anti-FBP1, anti-PD-L1 and anti-pSTAT3 (Y705) antibodies of TMA (n=35) tissue sections. (B) Heat map showing the staining index of FBP1, PD-L1 and pSTAT3 (Y705) in TMA. (C) Correlation analysis of the staining index for expression of FBP1 and PD-L1 in specimens of PDAC patients (n=35). Spearman's rank correlation coefficient and *P* values are shown. (D, E) Correlation analysis of mRNA level between PD-L1 and FBP1 in patient specimens of PRAD (n=497) and BLCA (n=407). Pearson product-moment correlation coefficient and *P* values are shown.

To assess the clinical relevance of FBP1 inhibition of PD-L1 expression, we examined the correlation between FBP1 and PD-L1 expression in PDAC patient samples by performing immunohistochemistry (IHC) on a TMA containing PDAC specimens from a cohort of 35 patients. IHC was evaluated by both staining intensity and percentage of positive cells. Representative IHC images displaying the high and low/no staining of FBP1 and PD-L1 are shown in Figure 2A. Further analysis showed that there exists a negative correlation between the level of FBP1 and the expression of PD-L1 among these patients (Spearman's rank correlation *r* = -0.5691, *P* < 0.0001) (Figures 2B and 2C). Meta-analysis of previously published data showed that expression of FBP1 and PD-L1 mRNA also inversely correlated in prostate adenocarcinoma (PRAD) and bladder urothelial carcinoma (BLCA) patient specimens (Figures 2D and 2E). Thus, these data suggest FBP1 may also negatively regulate PD-L1 expression in patients of different cancer types.

### FBP1 interacts with STAT3, a transcriptional regulator of PD-L1

**Figure 3:**
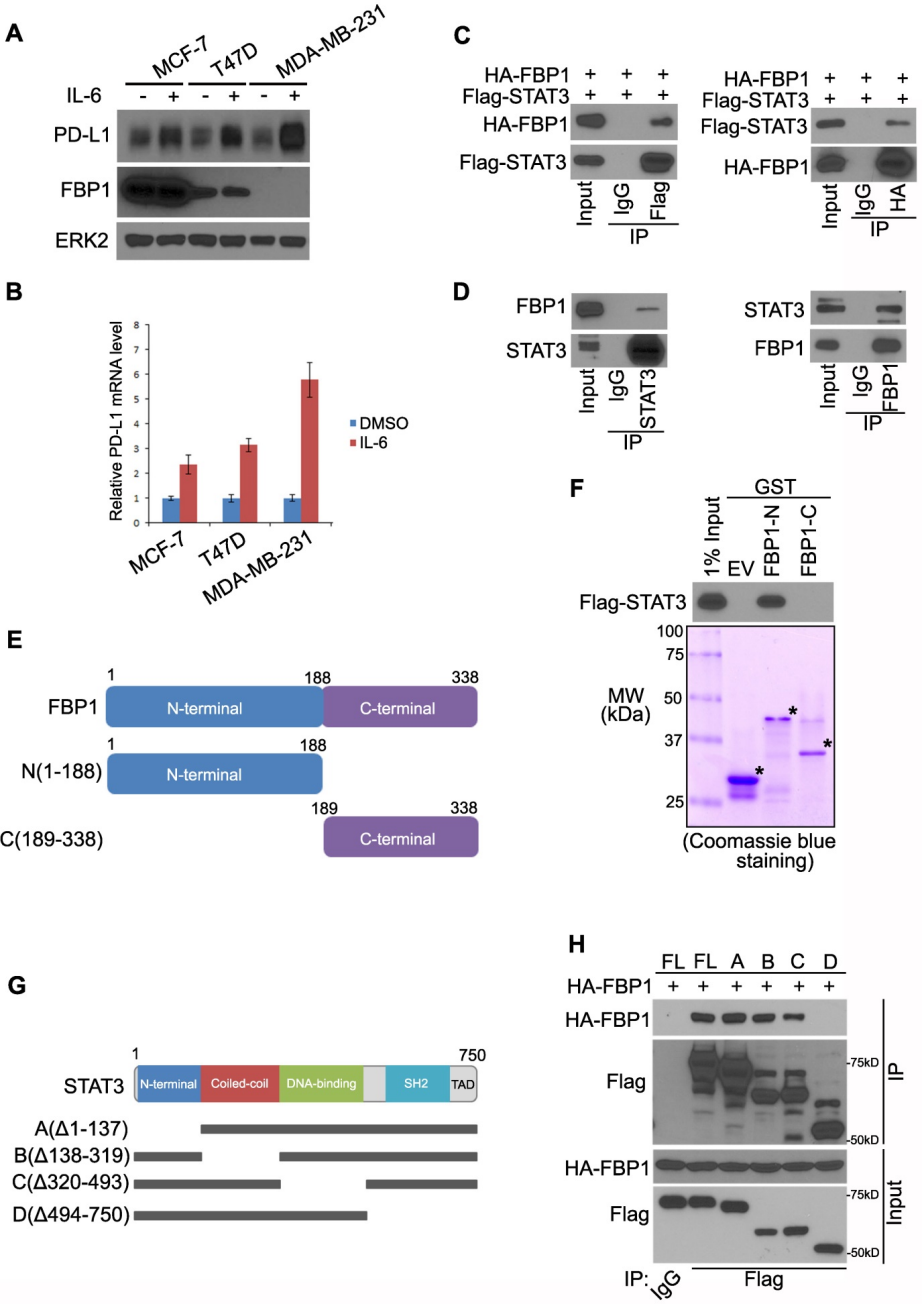
** FBP1 interacts with STAT3, a transcriptional regulator of PD-L1.** (A, B) MCF7, T47D and MDA-MB-231 cells were treated with IL-6 (200 ng) or PBS. 48 hours after treatment cells were harvested for western blot (A) and quantitative RT-PCR (B). All data are shown as mean values ± SD (n=3). (C, D) Western blot analysis of ectopically expressed HA-FBP1 and FLAG-STAT3 reciprocally immunoprecipitated by anti-HA and anti-FLAG in 293T cells (C), and endogenous FBP1 and STAT3 proteins reciprocally immunoprecipitated by anti-FBP1 and anti-STAT3 in MIA PaCa-2 cells (D). (E) Schematic diagram depicting two GST-FBP1 recombinant proteins. (F) Western blot analysis of Flag-STAT3 proteins in 293T whole cell lysate pulled down by GST or GST-FBP1 recombinant proteins. (G) Schematic diagram of a set of STAT3 mutant proteins. (H) Western blot analysis of ectopically expressed HA-FBP1 proteins in 293T whole cell lysate pulled down by FLAG or FALG-STAT3 WT and mutant proteins.

It has been reported that PD-L1 is upregulated by interleukin 6 (IL-6) through STAT3 signaling 38. We sought to determine whether FBP1 expression affects this process. As expected, IL-6 treatment induced upregulation of PD-L1 at both protein and mRNA level in three different cell lines including MCF-7, T47D, and MDA-MB-231, although IL-6 treatment had no effect on FBP1 protein expression (Figures 3A and 3B). Notably, we found that compared to FBP1 highly expressed cell lines MCF-7 and T47D, PD-L1 induction by IL-6 was much greater in MDA-MB-231 cells in which FBP1 was barely detectable (Figures 3A and 3B), suggesting that FBP1 might negatively influence the regulation of PD-L1 via the IL-6-STAT3 signaling pathway. To test this hypothesis, we first examined whether FBP1 interacts with STAT3. To this end, we performed co-immunoprecipitation (co-IP) assays. Reciprocal co-IP studies showed that ectopically expressed FBP1 formed a protein complex with STAT3 (Figure 3C). We further confirmed that FBP1-STAT3 interaction also occurs at the endogenous level in the cytoplasm (Figures 3D and S1A).

To define which domain of FBP1 mediates its interaction with STAT3, we constructed glutathione S-transferase (GST) expression plasmids for two truncatedFBP1 recombinant proteins (Figure 3E). GST pulldown assay demonstrated that GST-FBP1 N-terminal fragment (amino acids 1-188), but not GST or GST-FBP1 C-terminal fragment (amino acids 189-338) interacted with STAT3 (Figure 3F). To determine which domain(s) of STAT3 are responsible for the binding with FBP1, we generated four mutant plasmids of STAT3 according to four well-characterized functional domains of STAT3 (Figure 3G). Co-IP assay indicated that the SH2 domain of STAT3 is necessary for the interaction between STAT3 and FBP1 (Figure 3H). These data indicate that FBP1 can interact with STAT3 at endogenous level.

### STAT3 phosphorylation induced by ionizing radiation and IL-6 diminishes FBP1-STAT3 interaction

**Figure 4:**
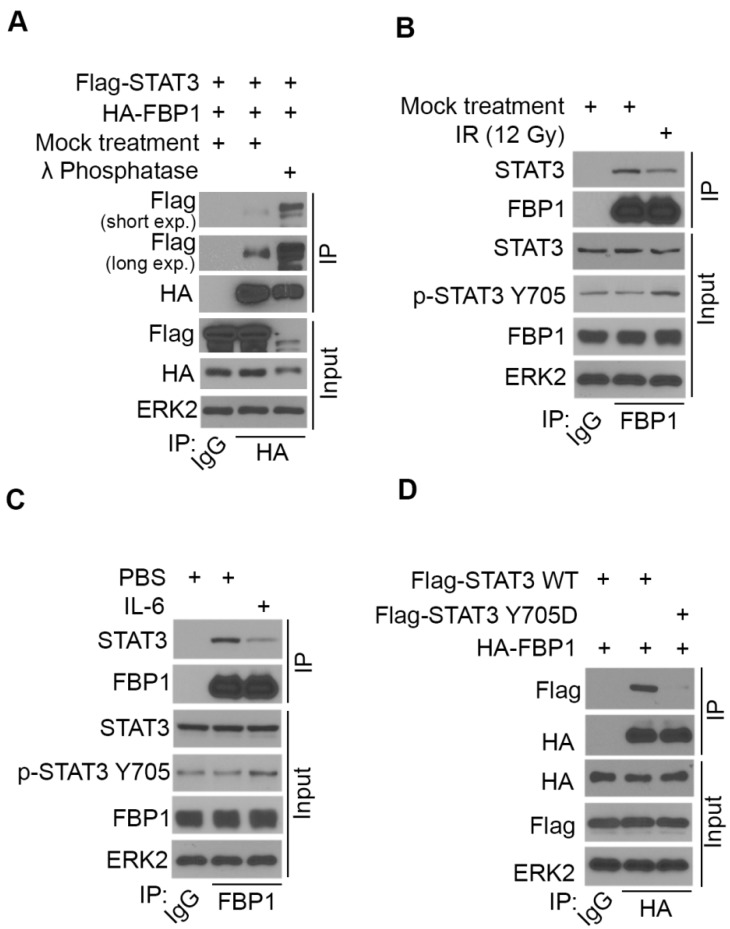
** STAT3 phosphorylation induced by ionizing radiation and IL-6 diminishes FBP1-STAT3 interaction.** (A) Western blot analysis of whole cell lysate and co-IP samples from 293T cells 24 hours after being transfected with the plasmids as indicated. (B) MIA PaCa-2 cells were treated with gamma radiation (12 Gy) or mock treatment. 24 hours later cells were harvested for western blot analysis of whole cell lysate and co-IP samples. (C) Western blot analysis of whole cell lysate and co-IP samples from MIA PaCa-2 cells 24 hours after being treated with IL-6 (200 ng) or mock treatment. (D) Western blot analysis of whole cell lysate and co-IP samples from 293T cells 24 hours after being transfected with the indicated plasmids.

It has been shown previously that induction of PD-L1 by IL-6 is highly associated with phosphorylation of STAT3 38, 39. We sought to determine whether phosphorylation of STAT3 affects its binding to FBP1. FBP1 and STAT3 were ectopically expressed in 293T cells and cell lysate was treated with λ protein phosphatase. We demonstrated that phosphatase treatment of cell lysate markedly enhanced FBP1-STAT3 interaction (Figure 4A), indicating protein phosphorylation inhibits FBP1 interaction with STAT3.

We have shown previously that ionizing radiation (IR) upregulates PD-L1 expression in various cancer cell lines 13. We examined whether IR affects STAT3 phosphorylation and FBP1-STAT3 interaction. While IR increased STAT3 phosphorylation at tyrosine 705 (Y705), IR dampened the interaction between FBP1 and STAT3 in MIA PaCa-2 cells (Figure 4B). Similarly, administration of IL-6 increased Y705 phosphorylation of STAT3, but decreased association of FBP1 with STAT3 (Figure 4C). Since phosphorylation of STAT3 at Y705 reduces the interaction between FBP1 and STAT3, we were interested to determine whether unphosphorylated Y705 in STAT3 is essential for its interaction with FBP1. We demonstrated that conversion of Y705 to aspartic acid (Y705D), a phosphor-mimicking mutant completely abolished FBP1 binding with STAT3 in 293T cells (Figure 4D). These data indicate that both IR and IL-6 can increase phosphorylation of STAT3, which prevents its interaction with FBP1 and that this effect is mediated by Y705 phosphorylation on STAT3.

To determine if there exists any association of pSTAT3 levels with FBP1 or PD-L1 in clinical patients, we also performed pSTAT3 (Y705) IHC on TMA of PDAC patients. Representative images of pSTAT3 IHC showed in Figure 2A displayed the high and low staining of pSTAT3. Further analysis showed that pSTAT3level is negatively correlated with FBP1 level (Spearman's rank correlation *r*= -0.7193, *P<* 0.0001) while pSTAT3 level is positively correlated with the expression of PD-L1 (Spearman's rank correlation *r*= 0.4704, *P<* 0.0001) (Figure S1B and S1C). These data suggest that signaling pathways influencing STAT3 phosphorylation can modulate FBP1 interaction with STAT3 and FBP1 regulation of PD-L1 expression.

### FBP1 represses PD-L1 expression via STAT3

**Figure 5:**
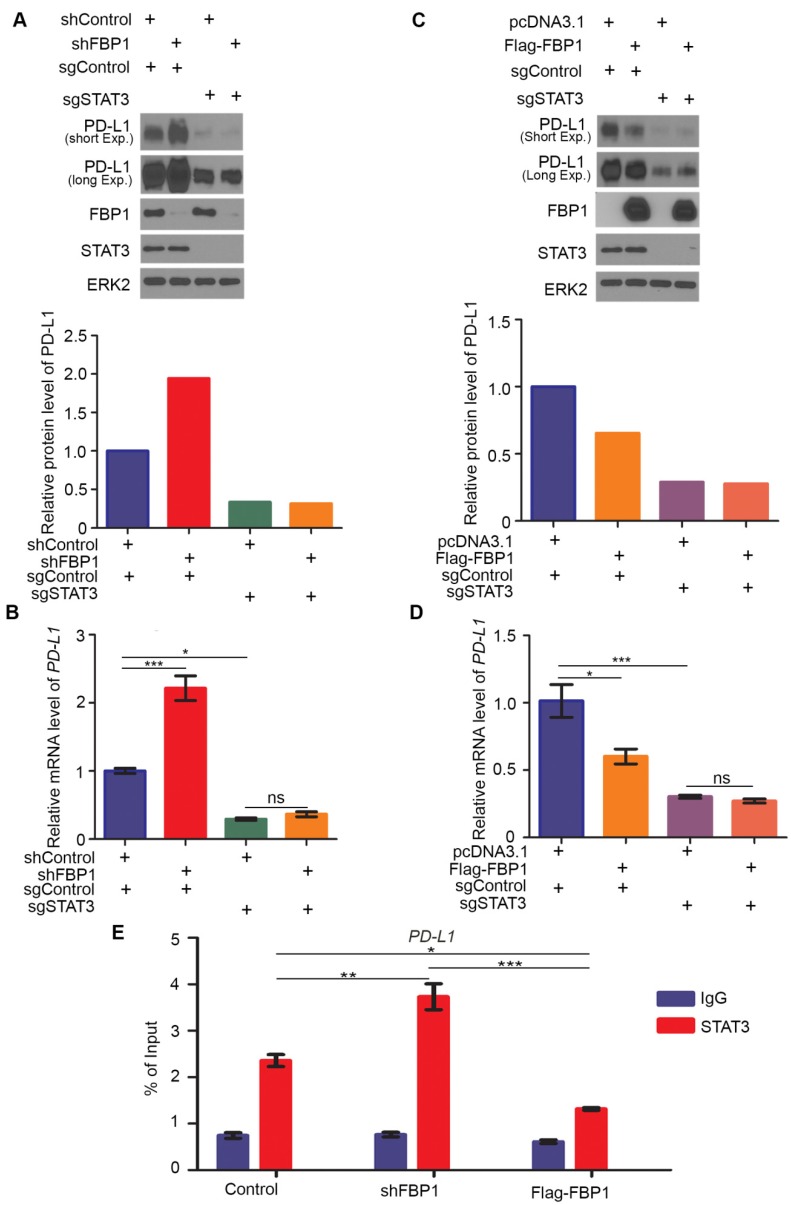
** FBP1 represses PD-L1 expression via STAT3.** (A, B) Western blot analysis of proteins (A) and quantitative RT-PCR measurement of the mRNA level of PD-L1 (B) in MIA PaCa-2 cells 48 hours after transfected with shRNA and sgRNA as indicated. All data are shown as mean values ± SD (n=3). ns, not significant, * *P* < 0.05, *** *P* < 0.001. (C, D) Western blot analysis of proteins (C) and mRNA level of PD-L1 (D) in MIA PaCa-2 cells 24 hours after transfected with plasmids and sgRNAs indicated. All data are shown as mean values ± SD (n=3). ns, not significant, * *P* < 0.05, *** *P* < 0.001. (E) ChIP-qPCR analysis of STAT3 binding at the *PD-L1* promotor in MIA PaCa-2 cells treated with shFBP1 or Flag-FBP1. All data are shown as mean values ± SD (n=3). *P < 0.05, ** *P* < 0.01, *** *P* < 0.01.

Our finding that FBP1 interacts with STAT3 suggests that FBP1 may regulate PD-L1 via STAT3. To test this hypothesis, we generated control and STAT3 knockout MIA PaCa-2 cells using CRISPR/Cas-9 (Figure 5A). We demonstrated that FBP1 KD upregulated PD-L1 expression at both protein and mRNA level in sgControl cells, but these effects were abolished in sgSTAT3 cells (Figures 5A and 5B). In contrast, FBP1 overexpression downregulated PD-L1 expression at both protein and mRNA level in sgControl cells, but such effects were abolished in sgSTAT3 cells (Figures 5C and 5D). Furthermore, chromatin immunoprecipitation-coupled quantitative PCR (ChIP-qPCR) analysis showed that STAT3 bound at the *PD-L1* gene locus (Figure 5E). Importantly, STAT3 occupancy at the *PD-L1* locus was increased by FBP1 KD but decreased by FBP1 overexpression (Figure 5E). Our data suggest that FBP1 inhibits PD-L1 expression in a way dependent on STAT3.

### Decreased expression of FBP1 promotes tumor growth and resistance to anti-PD-L1 treatment

**Figure 6:**
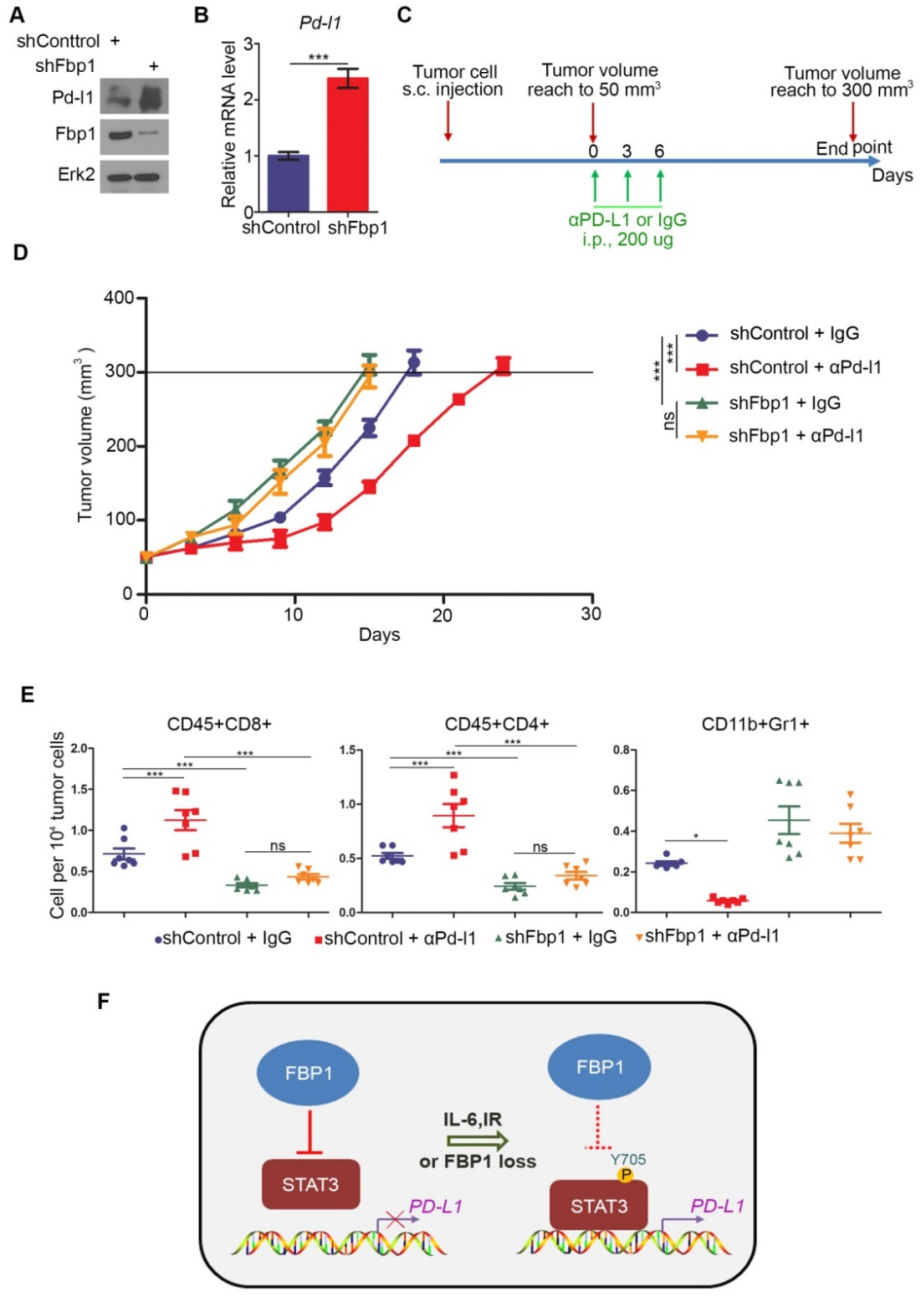
** Decreased expression of Fbp1 promotes tumor growth and resistance to anti-Pd-l1 treatment.** (A, B) PTEN-CaP8 murine prostate cancer cells were infected with lentivirus expressing control or Fbp1-specific shRNA. Cells were harvested for western blots (A) and quantitative RT-PCR (B). All data are shown as mean values ± SD (n=3), *** *P* < 0.001. (C-E) PTEN-CaP8 murine prostate cancer cells (5×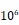 ) were infected with lentivirus as in (A) and injected subcutaneously into C57BL/6 mice (n=8/group). Mice were treated with anti-Pd-l1 (200 µg) or non-specific IgG when the allografts reached 50 mm³ and the treatment was repeated 3 days and 6 days later. Mice were euthanized when the tumor volume reached 300 mm^3^ (C). At the end of treatment, tumor volume was calculated by the formula L × W^2^ × 0.5 (D). The number of infiltrated T and myeloid cells in tumors was analyzed by FACS (E). All data are shown as mean values ± SD (n=8), ns, not significant, * *P* < 0.05, *** *P* < 0.001. (F) A hypothetical model depicting FBP1 repression of PD-L1 expression via the competitive interaction with STAT3. FBP1 loss, ionizing radiation or Y705 phosphorylation of STAT3 induced by IL-6 dampens the interaction between FBP1 and STAT3, thereby abolishing FBP1-mediated inhibition of PD-L1 expression and cancer progression.

Next, we examined how FBP1 affects tumor immunity. We knocked down Fbp1 in PTEN-CaP8 murine prostate cancer cells and Fbp1 knockdown significantly increased Pd-l1 expression at both mRNA and protein level in these cells (Figures 6A and 6B). We injected control or Fbp1 stable knockdown PTEN-CaP8 cells into immune-proficient C57BL/6 mice and mice were treated with control IgG or anti-Pd-l1 antibody when tumors reached the average size of 50 mm^3^ (Figure 6C). We demonstrated that knockdown of Fbp1 promoted the tumor growth (Figure 6D). To our surprise, while Fbp1-deficient cells expressed much more Pd-l1 proteins compared to control cells (Figure 6A), Fbp1-deficient tumors were more resistant to anti-Pd-l1 treatment than the control tumors (Figure 6D). Fractionation and cellular localization assays showed that there was substantial amount of Pd-l1 protein localized in the nucleus (Figures S1D and S1E). It has been shown previously that in addition to the immune checkpoint function, PD-L1 can also execute multiple intracellular functions, such as inducing activation of the mTOR-AKT pathway^40^, facilitating glucose metabolism^41^, ^42^ and acting as an RNA binding protein to inhibit DNA damage-related protein degradation^43^. Therefore, it is possible that nuclear PD-L1 in Fbp1 knockdown cells may acquire certain cellular functions, which provides a plausible explanation for the anti-PD-L1 therapy resistance observed in Fbp1 knockdown tumors in mice, but this notion warrants further investigation.

FACS analysis showed that knockdown of Fbp1 significantly decreased tumor infiltration of CD45^+^CD8^+^ T and CD45^+^CD4^+^ T cells, but increased the infiltration of CD11b^+^Gr1^+^ myeloid-derived suppressor cells (MDSCs) (Figure 6E). Furthermore, co-administration of Fbp1 knockdown and anti-pd-l1 antibody additively increased the tumor infiltration of CD45^+^CD8^+^ T and CD45^+^CD4^+^ T cells and a substantial reduction of CD11b^+^Gr1^+^ MDSCs (Figure 6E). These data indicate that knockdown of Fbp1 promotes prostate tumor growth and resistance to anti-Pd-l1 treatment in mice.

## Discussion

The major finding of the current study is the identification of a previously unrecognized molecular mechanism that causes cancer immune evasion and cancer progression. FBP1, a putative tumor suppressor, has been shown previously to inhibit tumor progression by inhibiting aerobic glycolysis and reducing the Warburg effect 30 and/or antagonizing the function of HIF in renal cancer 31. Here we demonstrated that FBP1 plays a pivotal role in regulating PD-L1 expression and cancer immunity in a manner independent of its enzymatic activity, thereby defining a new role of FBP1 in regulating the process of tumor progression. We found that FBP1 downregulates PD-L1 expression through the interaction with STAT3 and that FBP1 inhibits STAT3-dependent PD-L1 expression. Mechanistically, we further showed that FBP1 competitively sequesters the unphosphorylated STAT3, significantly decreases STAT3 occupancy on the genomic locus of *PD-L1* gene, and downregulates the expression of PD-L1. In contrast, ionizing radiation or IL-6 treatment increases Y705 phosphorylation of STAT3 and impairs the interaction between FBP1 and STAT3, thereby diminishing the inhibitory effect of FBP1 on PD-L1 expression (Figure 6F). These findings not only help us explain our observation that FBP1 downregulates the protein and mRNA level of PD-L1 in multiple cancer cell lines, but also provides a mechanistic explanation for the immune evasion caused by radiotherapy or inflammatory response in clinic. More importantly, we provide evidence showing that it is possibly a pervasive regulatory mechanism commonly occurring in different cancer types. Given that upregulation of PD-L1 expression in antigen-presenting cells (APCs) is also very important for cancer immune evasion, whether the FBP1-involved mechanism accounts for this upregulation in a paracrine dependent fashion warrants further investigation.

As a rate-limiting enzyme in the process of gluconeogenesis, FBP1 converts fructose-1,6-biphosphate into fructose-6-phophate and inorganic phosphate 34. Previous studies have showed that FBP1 is often downregulated in multiple types of solid tumors 28, 31, 44. The mechanism of the downregulation may associate with DNA hypermethylation of its promoter and copy number loss 28, 45, histone deacetylation due to the deregulation of HDACs 33 or the post-transcriptional modification mediated by MAGE-TRIM28 leading to the degradation of FBP1 in cancer cells 46. Importantly, the downregulation of FBP1 often links to the Warburg effect which is a common metabolic character of many solid tumors, including PDAC 34. In this study, we demonstrated that FBP1 inhibits PD-L1 expression via interacting with the STAT3 pathway. Consistent with the finding that FBP1 is often downregulated in human cancers, we provide compelling evidence that loss of FBP1 contributes to immune evasion of malignant tumors. Thus, our findings define a new role of FBP1 in tumor suppression.

STAT3 plays an important role in wound healing and tissue repair in normal tissues 47. However, excessive activation of STAT3 in malignant tumors results in inflammation-driven repair that promotes drug resistance or tumor progression 48-51. Previous studies have reported that activation of STAT3 or the IL-6-JAK-STAT3 signaling cascade in cancer cells promotes oncogenesis 52-55. Further studies show that STAT3 induces immunosuppression in cancer by upregulating PD-L1 and overexpression of PD-L1 significantly associates with the level of phosphorylated STAT3 38, 39. In the current study, we demonstrated that FBP1 interacts with STAT3 and inhibits the recruitment of STAT3 to the *PD-L1* gene locus. This inhibitory effect is attenuated by IL-6 treatment or ionizing radiation due to increased Y705 phosphorylation of STAT3. Our findings reveal FBP1 as an upstream inhibitor of STAT3 and uncover a new mechanism that regulates the function of STAT3 in cancer. The significance of these findings is further accentuated by our observations that expression of FBP1 and PD-L1 is negatively corrected in cell lines of multiple cancer types and PDAC patient specimens.

In summary, we discover a new role of FBP1 in antagonizing STAT3-dependent expression of PD-L1 and the mechanism through which FBP1 loss in human cancer cells contributes to the upregulation of PD-L1 and resistance to anti-PD-L1 treatment. This finding suggests that loss of FBP1 loss may be involved in immune evasion in human cancers although the direct evidences linking FBP1 expression to immune responses in patients is currently lacking. Further investigation of this mechanism in clinic could lead to define new strategies to improve the patient response to anti-PD-1/PD-L1 therapy in clinic.
