# Supplementary material for: Fructose-1,6-bisphosphatase loss modulates STAT3-dependent expression of PD-L1 and cancer immunity
Source: Theranostics. 2020 Jan 1;10(3):1033–45. doi: 10.7150/thno.38137 (PMC6956820; doi:10.7150/thno.38137)
Supplement: Supplementary file 1 — Supplementary tables S1-S2 and figures. [file thnov10p1033s1.pdf]

Supplementary Information

Fructose-1,6-bisphosphatase loss modulates STAT3-dependent expression of PD-L1 and cancer immunity

Bo Wang, Yingke Zhou, Jun Zhang, Xin Jin, Heshui Wu and Haojie Huang

Supplementary Table S1. Sequences of primers for RT-qPCR and ChIP-qPCR.

**For RT-qPCR (Human)**		
**Gene**	**Forward**	**Reverse**
GAPDH	ACCCAGAAGACTGTG GATGG	TTCAGCTCAGGGATGA CCTT
FBP1	ACATCGATTGCCTTGT GTCC	CCACCAAAATGAACTC CCCG
PD-L1	GGTGCCGACTACAAG CGAAT	AGCCCTCAGCCTGACA TGTC
**For RT-qPCR (Mouse)**		
**Gene**	**Forward**	**Reverse**
Gapdh	AGGTTGTCTCCTGCG ACTTCA	GGGTGGTCCAGGGTTT CTTACT
Pd-l1	AATGCTGCCCTTCAG ATCAC	ATAACCCTCGGCCTGA CATA
**For ChIP-qPCR (Human)**		
**Gene Locus**	**Forward**	**Reverse**
PD-L1	GGC GTT GGA CTT TCC TGA	CAA GGT GCG TTC AGA TGT TG

Supplementary Table 2. Sequences of shRNA and sgRNA

**Human**		
**Gene**	**Sequence**	
shFBP1-1	5′-CCGGCCTTGATGGATCTTCCAACATCTCGAGATGTTGGAAGATCCATCAAGGTTTTTG-3′	
shFBP1-2	5′-CCGGCGACCTGGTTATGAACATGTTCTCGAGAACATGTTCATAACCAGGTCGTTTTTG-3′	
SgSTAT3	**Forward:**	**Reverse:**
	CACCGAGATTGCCCGGATTGTGGCC	AAACGGCCACAATCCGGGCAATCTC
**Mouse**		
**Gene**	**Sequence**	
shFbp1	5′-CCGGCATAGCTTATGTCATGGAGAACTCGAGTTCTCCATGACATAAGCTATGTTTTTG-3′	

Supplementary Figure

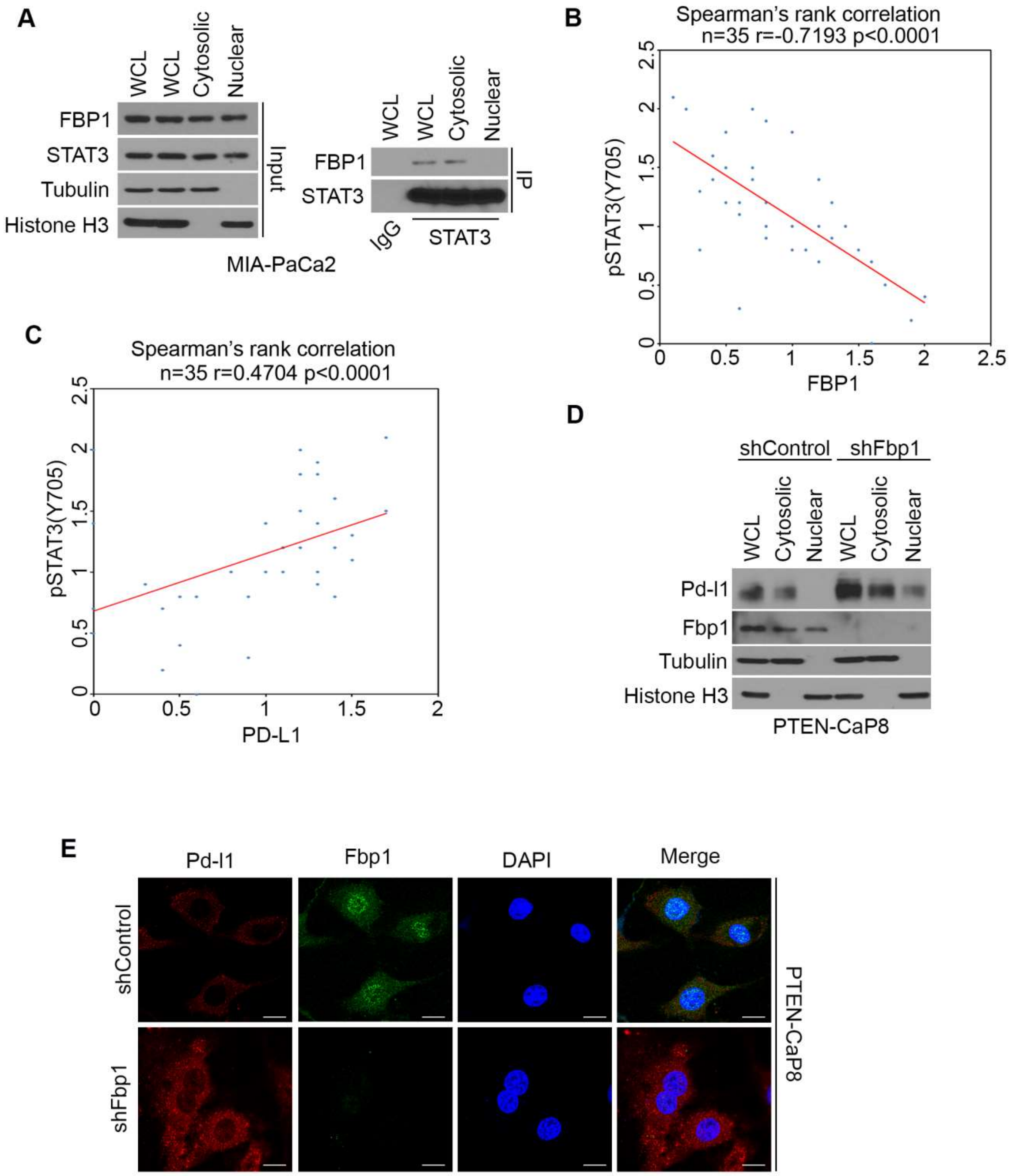

Figure S1. FBP1 interacts with STAT3 in the cytoplasm and knocking down of Fbp1 in murine PTEN-CaP8 cells changes Pd-l1 subcellular localization

(A) Western blot analysis of whole cell lysate (WCL), cytosolic fractionation and nuclear fractionation and co-immunoprecipitated endogenous FBP1 and STAT3 in Mia PaCa-2 cells.

(B) Correlation analysis of the staining index for expression of FBP1 and pSTAT3 (Y705) in specimens of PDAC patients (n=35). Spearman's rank correlation coefficient and *P* values are shown.

(C) Correlation analysis of the staining index for expression of pSTAT3 (Y705) and PD-L1 in specimens of PDAC patients (n=35). Spearman's rank correlation coefficient and *P* values are shown.

(D) Western blot analysis of whole cell lysate (WCL), cytosolic fractionation and nuclear fractionation in shControl and Fbp1 knocking down PTEN-CaP8 cells.

(E) PTEN-CaP8 cells were infected with lentivirus expressing indicated shRNA. The expression and subcellular localization of Fbp1 and Pd-l1 were examined using fluorescent cytochemistry (IFC).
